# Psychometric properties of the satisfaction with life scale (SWLS) in Iranian infertile women 

**Published:** 2016-01

**Authors:** Saman Maroufizadeh, Azadeh Ghaheri, Reza Omani Samani, Zahra Ezabadi

**Affiliations:** *Department of Epidemiology and Reproductive Health, Reproductive Epidemiology Research Center, Royan Institute for Reproductive Biomedicine, ACECR, Tehran, Iran.*

**Keywords:** *Infertility*, *Satisfaction with Life Scale*, *Psychometric properties*

## Abstract

**Background:**

Infertility is a global public health issue and may adversely affect life satisfaction. One of the most widely instruments used to assess life satisfaction is the Satisfaction with Life Scale (SWLS).

**Objective:**

The objective of this study was to examine the psychometric properties of the SWLS in Iranian infertile women.

**Materials and Methods:**

In this descriptive cross-sectional study, a total of 125 infertile women referring to Royan Institute in Tehran were selected by convenience sampling method. The participants were administered the SWLS, the Hospital Anxiety and Depression Scale (HADS), and a demographic questionnaire. The psychometric properties of the SWLS were examined: construct validity using confirmatory factor analysis (CFA), reliability using Cronbach’s alpha and convergent validity by examining the relationship with HADS.

**Result:**

Results of the CFA indicated that a single-factor model provides a good fit to the data (χ^2^/df= 1.58; GFI= 0.975; CFI= 0.995; NFI= 0.985; RMSEA= 0.069 and SRMR= 0.027). The Cronbach’s alpha coefficient for SWLS was 0.887. Significant negative correlations were found between SWLS and HADS scores for anxiety (r= -0.410) and depression (r= -0.434), indicating an acceptable convergent validity.

**Conclusion:**

The SWLS has adequate psychometric properties for assessing life satisfaction in Iranian infertile women.

## Introduction

Infertility has been recognized as a public health issue worldwide by the World Health Organization (WHO) and affects approximately 10-15% of reproductive-aged couple (1). A growing body of research demonstrates a high incidence of negative reactions to infertility and its treatment that impact on global life satisfaction and subjective well-being (2, 3). For instance, infertility is negatively associated with depression, anxiety, self-esteem, marital instability, social isolation and deprivation, and stress and tension in the relationship with their partner (2-4). Due to this impact, nowadays health-systems not only focus on morbidity and mortality, but also have impact on subjective well-being and quality of life. Subjective well-being includes two major components: an affective component, which is usually further divided into positive affect and negative affect, and a cognitive component, which is referred to as life satisfaction (5, 6).

The Satisfaction with Life Scale (SWLS) is one of the most widely used instruments for measuring the level of life satisfaction (7). It is a self-report questionnaire developed by Diener *et al*. The scale is a short and easy to use questionnaire and usually requires only about one minute of a respondent's time (7).

The SWLS has been used in various cultures and populations, and has been translated into various languages (8). A large body of research has also examined the psychometric properties of the SWLS (8-10). The exploratory and confirmatory factor analyses have supported a unidimensional structure of the SWLS as it was conceptualized by Diener *et al* (8-10). According to Diener and co-workers, the SWLS has been demonstrated to have strong internal consistency and moderate temporal stability with Cronbach’s alpha of 0.87 and 2-month test-retest reliability of 0.82 (7). Other researchers have reported similar results (8). The SWLS also has acceptable convergent validity; it is related to, but still separate from, constructs such as anxiety, depression, happiness, self-esteem, negative and positive effect, as well as psychological distress (8). To the best of our knowledge, no studies were found evaluating reliability and validity of the SWLS in infertile women, especially in Iran. Therefore, the present study aimed to examine the psychometrics properties of the SWLS in Iranian infertile women undergoing IVF treatments. In addition, the association between life satisfaction and demographic characteristics were examined.

## Materials and methods

**Study design and participants**

This cross sectional study was conducted in Royan Institute, Tehran, Iran from December 2013 to February 2014. The sample was selected via convenience sampling method and it was determined according to a subject to item ratio of at least 10:1 recommended by Nunnally for factor analysis (11). Thus, a total of 125 infertile women undergoing IVF were participated in the study. The eligibility criteria for this study were as follows: (a) women in a heterosexual marriage; (b) 18 years and older; (c) a diagnosis of infertility; and (d) ability to read and write in Persian. This study was approved by the Ethics Committee of Royan Institute and written informed consent was obtained from all participants.

**Instruments**

**Satisfaction with Life Scale (SWLS)**

The Satisfaction with Life Scale (SWLS) is a short 5-item instrument designed to measure global cognitive judgments of satisfaction with one's life. Each item scored on a 7-point Likert scale, ranging from 1 (strongly disagree) to 7 (strongly agree). Scale scores range from 5-35, with higher scores indicating greater life satisfaction (7). Scores are categorized as extremely satisfied (31-35), satisfied (26-30), slightly satisfied (21-25), neutral (20), slightly dissatisfied (15-19), dissatisfied (10-14), and extremely dissatisfied (5-9). The Persian version of SWLS available at: http://internal.psychology. illinois.edu/~ediener/SWLS.html was provided by Dr. Diener.

**Hospital Anxiety and Depression Scale (HADS)**

The HADS is a 14-item self-report inventory and composed of two subscales: Anxiety (HADS-A) and Depression (HADS-D). Both subscales of HADS consist of 7 items with each item scored on a 4-point Likert scale, ranging from 0-3. Subscale scores range from 0-21, with higher scores indicating higher level of anxiety and depression, respectively (12). The Persian version of HADS was used in the present study and it has been shown to have satisfactory reliability and validity (13). The Cronbach’s alpha coefficient for HADS-A and HADS-D in the present study were 0.846 and 0.703, respectively.

**Statistical analysis**

A confirmatory factor analysis (CFA) was conducted to examine the factor structure of the SWLS using covariance matrices and the maximum-likelihood estimation method. Goodness of fit of model was assessed using the chi-square (χ^2^), relative chi-square (χ^2^/df), the goodness of fit index (GFI), the comparative fit index (CFI), the normed fit index (NFI), the root mean square error of approximation (RMSEA), and the standardized root mean square residual (SRMR). The χ^2^ statistic is the most common methods of evaluating goodness of fit, but it is highly sensitive to sample size. An alternate evaluation of the χ^2^ statistic is to examine the relative chi-square (χ^2^/df) for the model (14). A χ^2^/df ratio of less than 2 is indicative of a good model fit (15). For other goodness of fit indexes, values indicative of good fit are GFI, CFI, and NFI >0.95, RMSEA <0.06 and SRMR <0.08 (16).

Internal consistency of the SWLS was examined using Cronbach’s alpha coefficient and corrected item-total correlations. Convergent validity of the SWLS was assessed by calculating Pearson correlation coefficients between the SWLS and HADS. Descriptive statistics for continuous variables were presented as mean±SD and for categorical variables as numbers (percentage). Moreover, one-way ANOVA and Pearson’s correlation coefficient were used to examine the relationship between SWLS scores and demographic characteristics. All preliminary analyses were performed using Statistical Package for the Social Sciences, version 16.0, SPSS Inc, Chicago, Illinois, USA (SPSS) and CFA was performed using Lisrel 8.80 (Scientific Software International, Inc., Lincolnwood, IL, USA). All statistical tests were two-tailed and p<0.05 was considered statistically significant.

## Results

**Participant Characteristics**

The socio-demographic and clinical characteristics of the participants are shown in table I. The mean age of participants was 31.23±5.81 yrs. Of the patients, the majority of them were male factor, 42.4% had a college or university degree, 40.8% had no failure in previous treatments, and 82.4% had no history of abortion. The mean duration of infertility was 6.45±4.54 and 78.4% of women had primary infertility. 

The mean total SWLS score was 23.65±6.70; all the participants were slightly satisfied with life. 

**Reliability and Item Analysis**

Cronbach’s alpha coefficient for assessing internal consistency of the SWLS was 0.887. All corrected item-total correlations were greater than the acceptable cut-off of 0.3 indicating each item was related to the overall scale. The inter-item correlations (data not shown) were also acceptable within the range of 0.492-0.824. The mean and standard deviation for each of the five items are also presented in table II.

**Table I:** Socio-demographic and clinical characteristics of the participants (n=125)

	**n (%)**
Age (years)	31.23 ± 5.81^a^
Duration of infertility (years)	6.45 ± 4.54^a^
Cause of infertility	
Male factor	56 (44.8)
Female factor	25 (20.0)
Both	21 (16.8)
Unexplained	23 (18.4)
Type of infertility	
Primary	98 (78.4)
Secondary	27 (21.6)
Educational level	
Primary	24 (19.2)
Secondary	48 (38.4)
University	53 (42.4)
Failure of previous treatment	
0	51 (40.8)
1	32 (25.6)
2	18 (14.4)
3	16 (12.8)
4	8 (6.4)
History of abortion	
0	103 (82.4)
1	14 (11.2)
2	8 (6.4)

a Values are shown in Mean±SD

**Table II:** Items Wording and Descriptive Statistics of the SWLS

**Item**	**Mean**	**SD**	**Corrected item total correlation**	**Alpha if item deleted**
In most ways my life is close to ideal	4.58	1.60	0.729	0.862
The conditions of my life are excellent	5.04	1.52	0.828	0.840
I am satisfied with my life	5.51	1.45	0.776	0.853
So far, I have gotten the important things I want in life	4.45	1.58	0.743	0.859
If I could live my life over, I would change almost nothing	4.07	1.88	0.600	0.899

**Convergent Validity**

To examine the convergent validity of the SWLS, Pearson correlation coefficients were calculated between SWLS and the HADS. As expected, the SWLS was significantly negatively correlated with the HADS-A (r= -0.410; p<0.001) and HADS-D (r= -0.434; p<0.001), indicating an acceptable convergent validity.

**Confirmatory Factor Analysis**

The CFA was used to determine the goodness of fit of the previously identified one-factor model. 

The goodness of fit indices revealed that the single-factor model was a good fit to the data (χ^2^=7.91, df=5, p=0.161; χ^2^/df=1.58; GFI=0.975; CFI=0.995; NFI=0.985; RMSEA=0.069 and SRMR=0.027). 

**Fig 1:**
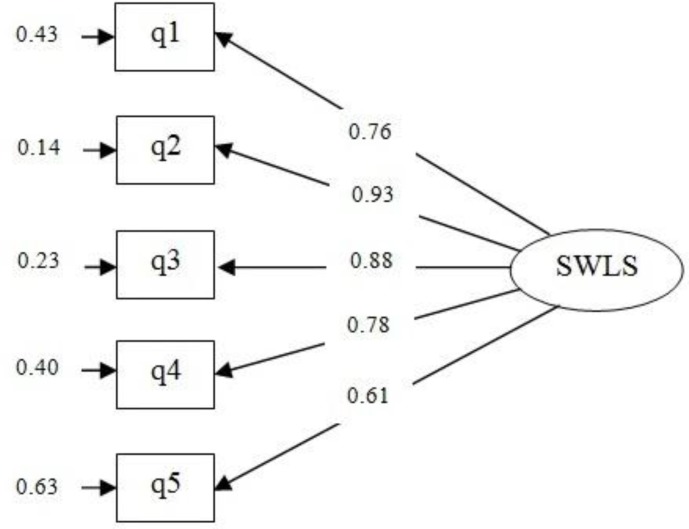
Confirmatory factor analysis of the single-factor model SWLS

Standardized factor loadings for single-factor model are shown in figure 1. All factor loadings were significant and in the expected direction, ranging from 0.61-0.93.

**Relationship of SWLS with Demographic Characteristics**

SWLS scores were uncorrelated with age (r=0.041; p=0.653) and durations of infertility (r=0.013; p=0.884). The relationships between SWLS and other demographic characteristics are shown in table III. Patients with two failures scored lower than other patients on SWLS, although the difference was not statistically significant (F(4, 120)=1.07; p=0.103). Scores also did not differ significantly by cause of infertility (F(3,121)=2.16; p=0.096), type of infertility (F(1,123)=0.03; p=0.852), educational level (F(2,122)=0.69; p=0.500), and history of abortion (F (2,122)=0.15; p=0.858).

**Table III:** Relationship of SWLS with Demographic Characteristics

	**SWLS**	**F Statistic**	**p-value** b
Cause of infertility		F (3,121)=2.16	0.096
Male factor	24.12±6.64^a^		
Female factor	25.68±5.99		
Both	22.76±6.88		
Unexplained	21.09±6.90		
Type of infertility		F (1,123)=0.03	0.852
Primary	23.59±6.85		
Secondary	23.86±6.25		
Educational level		F (2,122)=0.69	0.500
Primary	24.25±6.53		
Secondary	22.75±6.90		
University	24.19±6.63		
Failure of previous treatment		F (4,120)=1.97	0.103
0	24.35±7.02		
1	23.59±6.10		
2	20.78±6.13		
3	22.56±7.64		
4	28.00±3.58		
History of abortion		F (2,122)=0.15	0.858
0	23.58±6.76		
1	24.50±6.09		
2	23.00±7.59		

a Values are shown in Mean±SD

b One-way ANOVA

## Discussion

The purpose of this study was to examine the psychometric properties of the persian version of the SWLS in infertile women. The SWLS was found to have high internal consistency, and the item-total and inter-item correlations were quite acceptable. This finding is quite consistent with previous studies that have reported high internal consistency for SWLS in clinical and population based samples (8, 9). 

The present study supported the single-factor structure of the SWLS in infertile women, which is consistent with previous studies (7-9). Our findings confirmed the expected negative relationship between SWLS and anxiety and depression. Infertile women with a high SWLS score had lower levels of anxiety or depression, and vice versa. Thus, healthcare professionals should consider infertile women’s psychological distress when examine and treat their infertility in order to optimize their life satisfaction. This finding is in line with previous studies and confirms the convergent validity of SWLS (8, 17).

Although the difference was not statistically significant, the patients with two failures, on average, reported lower life satisfaction than the other patients. This finding suggests that a comprehensive approach including psychological intervention is necessary for women with two failures to improve the life satisfaction. 

In a study conducted by Maroufizadeh *et al*, also anxiety and depression were worst in women experiencing two and one failure, respectively (18). Furthermore, there was not found any significant relationship between SWLS and other demographic characteristics (age, duration of infertility, cause of infertility, type of infertility, educational level, and history of abortion). 

Our results indicated that women undergoing infertility treatments were slightly satisfied with their lives (score 21-25). It is possible, therefore, that family, friends and society supports can improve these women’s life satisfaction.

This study has a few limitations. First, because of the practical reasons, only the infertile women included in the study and their partners were not participated. Second, only the patients undergoing IVF treatment included in the study and the patients in the pre-treatment, diagnostic phase or other Assisted Reproductive Technology were not investigated. So, generalization of the results may be affected by the sample. Third, the test-retest reliability was not examined in this study. 

On the other hand, to our knowledge, this is the first study examining psychometric properties of the SWLS in a sample of infertile women.

In conclusion, the persian version of the SWLS has acceptable psychometric properties for measuring life satisfaction in infertile women. However, further psychometric studies in diverse populations of infertile people are needed.
